# The Ebola virus disease outbreak in Tonkolili district, Sierra Leone: a retrospective analysis of the Viral Haemorrhagic Fever surveillance system, July 2014–June 2015

**DOI:** 10.1017/S0950268819000177

**Published:** 2019-02-26

**Authors:** Alessandro Miglietta, Angelo Solimini, Ghyslaine Bruna Djeunang Dongho, Carla Montesano, Giovanni Rezza, Vincenzo Vullo, Vittorio Colizzi, Gianluca Russo

**Affiliations:** 1 Epidemiology and Preventive Medicine Units, Central Tuscany Health Authority, Florence, Italy; 2 Department of Public Health and Infectious Diseases, Faculty of Medicine and Pharmacy, ‘Sapienza’ University of Rome, Rome, Italy; 3 Department of Biology, University of Rome ‘Tor Vergata’, Rome, Italy; 4 Department of Infectious Diseases, Istituto Superiore di Sanità, Rome, Italy

**Keywords:** Analysis of data, Ebola virus, outbreaks, surveillance system

## Abstract

In Sierra Leone, the Ebola virus disease (EVD) outbreak occurred with substantial differences between districts with someone even not affected. To monitor the epidemic, a community event-based surveillance system was set up, collecting data into the Viral Haemorrhagic Fever (VHF) database. We analysed the VHF database of Tonkolili district to describe the epidemiology of the EVD outbreak during July 2014–June 2015 (data availability). Multivariable analysis was used to identify risk factors for EVD, fatal EVD and barriers to healthcare access, by comparing EVD-positive *vs.* EVD-negative cases. Key-performance indicators for EVD response were also measured. Overall, 454 EVD-positive cases were reported. At multivariable analysis, the odds of EVD was higher among those reporting contacts with an EVD-positive/suspected case (odds ratio (OR) 2.47; 95% confidence interval (CI) 2.44–2.50; *P* < 0.01) and those attending funeral (OR 1.02; 95% CI 1.01–1.04; *P* < 0.01). EVD cases from Kunike chiefdom had a lower odds of death (OR 0.22; 95% CI 0.08–0.44; *P* < 0.01) and were also more likely to be hospitalised (OR 2.34; 95% CI 1.23–4.57; *P* < 0.05). Only 25.1% of alerts were generated within 1 day from symptom onset. EVD preparedness and response plans for Tonkolili should include social-mobilisation activities targeting Ebola/knowledge-attitudes-practice during funeral attendance, to avoid contact with suspected cases and to increase awareness on EVD symptoms, in order to reduce delays between symptom onset to alert generation and consequently improve the outbreak-response promptness.

## Introduction

Ebola virus disease (EVD) is a life-threatening illness due to Ebola virus (EBOV) [1]. Human EBOV infection is accidental, through direct contact with infected animals involved in the epi-enzootic viral cycle [2]. Once entered into the humans’ transmission chain, EBOV spreads through direct or indirect contact with bodily fluids of symptomatic or deceased EVD persons [3]. The EVD incubation period is 2–21 days, and humans are infectious only when symptomatic, but the long-lasting persistence of EBOV-RNA in semen of male EVD survivors raised concerns on sexual transmission after EVD recovery [4].

The 2013–2016, West-African EVD epidemic was the largest, deadliest and most complex outbreak since EBOV was discovered in 1976 [5]. Differently from other outbreaks, the West-Africa's 2013–2016 EVD outbreak involved urban impoverished and overcrowded areas of multiple countries, as well as isolated villages (thanks to recent improvement of connections in these countries), causing more cases and deaths than all the 23 previous EVD outbreaks combined. It began in Guinea in late 2013, spreading to Liberia and Sierra Leone during early 2014 [6], with smaller linked outbreaks in Nigeria, Mali and Senegal; imported cases were also reported in Europe and USA, with secondary infections of health care workers (HCWs), but without further spread [7]. On 8 August 2014, the World Health Organization (WHO) declared the West-African EVD outbreak a Public Health Emergency of International Concern (PHEIC) [8].

First EVD cases in Sierra Leone were reported from Kailahun district in May 2014 [9] and the highest incidence at national level was reached during August–December 2014, after which it declined as a result of the rapid scale-up of isolation, treatment and safe burial capacity in the country [10]. When the PHEIC status was declared over (29 March 2016) [11], the three West-African countries globally accounted for 28 610 EVD cases and 11 308 related deaths, with Sierra Leone reporting the highest burden of disease (14 124 cases and 3955 related deaths) [3]. The outbreak had devastating impact on the health systems and pronounced socio-economic effects in the three West-African countries [12].

Located in the centre of Sierra Leone, Tonkolili district borders with seven districts, is divided in 11 chiefdoms with a population of 455 383 people (Fig. S1) [13].

Although Tonkolili accounted for 5.2% of EVD cases and 4.5% of related deaths reported in the country [14], during the epidemic it experienced for three times the identification of new EVD cases (one linked to a transmission chain of Freetown and two with unknown origin) after having been declared as EVD-free district, with the last case reported on 14 January 2016, after that the WHO declared the end of Ebola transmission in the country (7 November 2015) [15, 16]. These recrudescences were of international concern and determined a change in the response practices in terms of number of contacts traced and number of staff rapidly deployed for the response [17].

To monitor the EVD epidemic, the Ministries of Health (MoH) of Sierra Leone, Guinea and Liberia, the Centers for Disease Control and Prevention (CDC) and the WHO, set up a community event-based surveillance (CEBS) system using the Epi Info Viral Haemorrhagic Fever (VHF) application (http://epiinfovhf.codeplex.com/) [18]. The system collects demographic, epidemiological, clinical and laboratory information on suspected EVD cases, and data allowing measuring the MoH and WHO key-performance indicators for EVD response [19]. The system is implemented at district level through the district Ebola response centre (DERC), with the support of WHO/CDC epidemiologists and the district health management team (DHMT) [20].

This study presents EVD surveillance data from the VHF database of Tonkolili district, Sierra Leone, with the principal objectives to describe the epidemiology of the EVD outbreak into the district during July 2014–June 2015 (data availability), to identify risk factors for EVD, fatal EVD and to assess barriers to healthcare access among EVD cases. Secondary objectives were to measure the MoH/WHO key-performance indicators for EVD response, and to present strategic analyses that can be performed from the VHF database, in order to guide decision making for national and international partners planning and implementing the response, also in terms of future preparedness.

## Methods

### Data collection: the EVD CEBS and the Epi Info VHF application

The EVD CEBS works by detecting alerts generated in the community and/or in healthcare facilities (HCF)/Ebola Treatment Centre/Unit (ETC/ETU) [21]. According to the WHO [22], an EVD alert is defined as: any person with illness onset of fever and no response to treatment of usual causes of fever in the area.

OR at least one of the following signs: bleeding, bloody diarrhoea, bleeding into urine

OR any sudden death.

Alerts are collected at the DERC through the Ebola-hotline (117) and screened by trained surveillance officers. Only if the alert meets the EVD definition of suspected/probable case, trained case investigators and/or contact tracers perform the case investigation using a standardised form. In parallel, a clinical sample is collected (blood for alive and oral swab for dead patients) and sent to the district's reference laboratory for confirmation through real-time reverse-transcriptase polymerase chain reaction (RT-PCR) test specific for EBOV [23].

Investigated alerts are daily reported to the WHO/CDC Epidemiologist (surveillance pillar) that also receives and matches the laboratory results in order to assign the final case classification. There are four final case classifications according to the WHO EVD case definition [22]:

SUSPECTED CASE: any person, alive or dead, suffering or having suffered from a sudden onset of high fever and having had contact with:

–a suspected, probable or confirmed Ebola case;

–a dead or sick animal (for Ebola)

OR: any person with sudden onset of high fever and at least three of the following symptoms:

headaches, vomiting, anorexia/loss of appetite, diarrhoea, lethargy, stomach pain, aching muscles or joints, difficulty swallowing, breathing difficulties, hiccup

OR: any person with inexplicable bleeding

OR: any sudden, inexplicable death.

PROBABLE CASE: any suspected case evaluated by a clinician

OR: any deceased suspected case (where it has not been possible to collect specimens for laboratory confirmation) having an epidemiological link with a confirmed case.

LABORATORY-CONFIRMED CASE (EVD+): any suspected or probably cases with a positive laboratory result. Laboratory-confirmed cases must test positive for the virus antigen, either by detection of virus RNA by RT-PCR, or by detection of IgM antibodies directed against Marburg or Ebola.

NON-CASE (EVD–): any suspected or probable case with a negative laboratory result. ‘Non-case’ showed no specific antibodies, RNA or specific detectable antigens.

Epidemiological, clinical and laboratory data are finally entered into the VHF database by a trained data-clerk and double checked with the WHO/CDC epidemiologist.

Alert's-related variables entered into the VHF database include demographic (e.g. gender, age, residence, occupation), epidemiological and clinical information, as the date of symptom onset, the clinical presentations, the outcome (and eventually date, place of death and of the burial), laboratory results and laboratory turnaround time, the number of contacts traced and whether the patient was hospitalised (and eventually date of hospitalisation) and was in a contacts tracing list. Finally, the following information on risk factors is collected: (1) direct contact with known or suspected/probable EVD case, including the date of exposure, (2) funeral attendance touching/carrying the body.

### Data analysis

We retrospectively analysed alerts reported into the VHF database of Tonkolili district during the period 1 July 2014–30 June 2015 (epi-week 27, 2014 to epi-week 26, 2015). Incidence, mortality and hospitalisation rates per 100 000 inhabitants were calculated using the 2015 population data (only available by chiefdom) provided by the DHMT of Tonkolili.

Risk factors for EVD+, fatal EVD+ and barriers to healthcare access among EVD+ cases were assessed through univariate (*χ*^2^) and multivariable analysis. Variables with a *P*-value <0.2 at the univariate analysis were included in a multivariable stepwise regression model, with a *P*-value >0.10 for backward elimination [24]. As dependent variables we used: EVD+ *vs*. EVD− cases to assess risk factors for EVD+; EVD+ dead *vs.* EVD+ alive cases to assess death's risk factors among EVD+ cases; hospitalised EVD+ *vs*. non-hospitalised EVD+ cases to evaluate barriers to healthcare access among EVD+ cases. For the hospitalisation and death analyses, we also assessed the symptoms (one *vs.* all the others) associated with these two outcomes, adjusting by age groups and gender.

Independent variables were: gender, age group (<6, 6–15, 16–30, >30years), chiefdom of residence (Gbonkolenken, Kafe Simiria, Kalansogoia, Kholifa Mabang, Kholifa Rowalla, Kunike, Kunike Barina, Malal Mara, Sambaya Bendugu, Tane, Yoni and ‘outside district’), occupation (HCW/other occupation), hospitalisation (yes/no), time interval from symptom onset to hospitalisation (⩾10; 3–9; ⩽2 days), clinical presentations (symptoms), and whether in the 21 days before symptom onset the patient attended a funeral touching the body and/or had contact with a confirmed/suspected/probable EVD case.

For EVD+ cases with a known exposition timing, we also calculated the incubation period.

Finally, the following MoH and WHO key-performance indicators for EVD response were measured:

1. Percentage of alert generated within 1 day from symptoms onset
2. Number and percentage of HCWs infected
3. Percentage of samples tested within 1 day of collection
4. Percentage of deaths buried within 1 day
5. Percentage of lives alert tested for EBOV
6. Percentage of deaths alert tested for EBOV
7. Percentage of reported community deaths that were tested for EBOV
8. Percentage of new confirmed cases from registered contacts
9. Number of hospitalised within 3 days from symptom onset
10. Reporting the case fatality rate among hospitalised EVD+ cases
11. Reporting the number of Ebola survivors by gender, age group, chiefdom
12. Reporting the number of contacts traced per EVD+ case

To evaluate failures of the surveillance system over the time and by place, the achievement of indicators was assessed by trimesters and chiefdom using a logistic regression model, adjusting by age group and gender. Indicators included in this analysis were numbers: 1, 3, 5, 6, 7, 9.

The others were excluded because close to 100% or not suitable for logistic regression analysis. Results were expressed in terms of odds ratios (ORs), with 95% confidence interval (95% CI). Statistical significance was set at *P*-value <0.05. R-software (version 3.3.1) was used for data analysis.

### Ethical approval

Ethical clearance was obtained from: Sierra Leone Ethics and Scientific Review Committee; MoH; Directorate of Policy, Planning and Information (DPPI). Informed consent was not requested because data were collected in the framework of a national surveillance system.

## Results

During the 1-year study period, 4550 alerts were reported and classified as follows: 454 (10%) EVD+, 3344 (73.5%) EVD–, 53 (1.2%) probable and 699 (15.4%) suspected cases (Table 1).

**Table 1.** Characteristics of the study population by Ebola virus disease case definition, Tonkolili district, Sierra Leone, 1 July 2014–30 June 2015 (*n* = 4550)

Characteristics		EVD+ (*n* = 454)	EVD− (*n* = 3344)	EVD suspected and probable (*n* = 752)^a^	Total (*n* = 4550)
		N (%)	N (%)	N (%)	N (%)
Gender	Male	197 (43.4)	1719 (51.4)	386 (51.3)	2302 (50.6)
	Female	257 (56.6)	1625 (48.6)	366 (48.7)	2248 (49.4)
Age group	<6 year	25 (5.5)	1188 (35.5)	226 (30.1)	1439 (31.6)
	6–15 years	52 (11.5)	208 (6.2)	60 (8.0)	320 (7.0)
	16–30 years	160 (35.2)	672 (20.1)	117 (15.6)	949 (20.9)
	>30 year	217 (47.8)	1276 (38.2)	349 (46.4)	1842 (40.5)
Chiefdom of residence	Gbonkolenken	76 (16.7)	625 (18.7)	99 (13.2)	800 (17.6)
	Kafe Simiria	5 (1.1)	107 (3.2)	37 (4.9)	149 (3.3)
	Kalansogoia	0 (0.0)	125 (3.7)	19 (2.5)	144 (3.2)
	Kholifa Mabang	3 (0.7)	108 (3.2)	26 (3.5)	137 (3.0)
	Kholifa Rowalla	126 (27.8)	656 (19.6)	142 (18.9)	924 (20.3)
	Kunike	77 (17.0)	291 (8.7)	102 (13.6)	470 (10.3)
	Kunike Barina	6 (1.3)	175 (5.2)	32 (4.3)	213 (4.7)
	Malal Mara	8 (1.8)	127 (3.8)	61 (8.1)	196 (4.3)
	Sambaya Bendugu	0 (0.0)	30 (0.99)	5 (0.7)	35 (0.8)
	Tane	51 (11.2)	223 (6.7)	51 (6.8)	325 (7.1)
	Yoni	93 (20.5)	668 (20.0)	145 (19.3)	906 (19.9)
	Outside districts	9 (2.0)	209 (6.3)	33 (4.4)	251 (5.5)
Occupation	Health care worker	11 (2.4)	23 (0.7)	19 (2.5)	53 (1.2)
	Other occupation	443 (97.6)	3321 (99.3)	733 (97.5)	4497 (98.8)
Hospitalised	Yes	303 (66.7)	1316 (39.4)	335 (44.5)	1954 (42.9)
	No	151 (33.3)	2028 (60.6)	417 (55.5)	2596 (57.1)
Type of alerts	Dead	93 (20.59	2355 (70.4)	451 (60.0)	2899 (63.7)
	Alive	361 (79.5)	989 (29.6)	301 (40.0)	1651 (36.3)

EVD+, Ebola laboratory-confirmed cases; EVD−, Ebola laboratory-excluded cases.

a EVD suspected cases = 699; EVD probable cases = 53.

Overall, 40.2% of alerts were from two chiefdoms (Kholifa Rowalla 20.3%; Yoni 19.9%), concerned people aged >30 years (40.5%) and dead persons (63.7%). HCWs represented the 1.2% (*n* = 53) of the study population; of them, 11 (20.8%) were EVD+. The distribution of EVD+ cases was proportioned between male (51.4%) and female (48.6%), whilst 35.5% of EVD+ alert was reported among those aged <6 years.

**Fig. 1.**
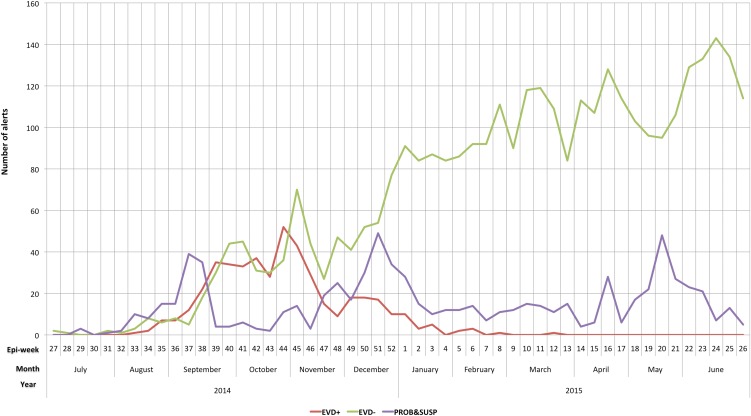
Number of alerts by epi-week, months, year and EVD case definition. Tonkolili, Sierra Leone, 1 July 2014–30 June 2015 (*n* = 4550). EVD+, Ebola laboratory-confirmed cases; EVD−, Ebola laboratory-excluded cases; PROB and SUSP, Ebola probable and suspected cases.

Figure 1 shows the number of alert by epi-week and case definition.

Alerts increased gradually with time: from six (0.1%) in July 2014, to 332 (7.3%) in October 2014, 531 (11.7%) in March 2015, reaching the peak in June 2015 (*n* = 609; 13.4%). The first EVD+ alert was a 14-year-old female from Kholifa Rowalla chiefdom (reported on 19 August 2014). In September 2014, 56 EVD+ additional cases were reported, with a district incidence rate (IR) of 147.6/100 000 inhabitants; then the EVD+ case count increased, with the higher IR during October (*n* = 152; IR = 400.5/100 000) and November 2014 (*n* = 149; IR = 392.6/100 000). After that, the EVD+ case count decreased continuously: 63 EVD+ cases (IR = 168.6/100 000) in December 2014, 24 (IR = 63.2/100 000) in January 2015 and six (IR = 15.8/100 000) in February 2015. The last recorded EVD+ case was imported from Western Area Rural district in Yoni chiefdom on 15 March 2015 (epi-week 12).

The average EVD+ R during the study period (Table 2) was 99.7/100 000 inhabitants (range: 0.0 in Kalansogoia and Sambaya Bendugu chiefdoms, to 209.0 in Kholifa Rowalla), while the average EVD+ mortality rate was 20.4/100 000 inhabitants (range: 0.0 in the chiefdoms of Kalansogoia, Kholifa Mabang and Sambaya Bendugu, to 48.1 in Kholifa Rowalla) and the average EVD+ hospitalisation rate 66.5/100 000 inhabitants (range: 8.1 in Kafe Simiria to 127.6 in Tane) with two-thirds of EVD+ cases hospitalised (*n* = 303/454; 66.7%).

**Table 2.** Incidence, mortality and hospitalisation rates (per 100 000 inhabitants) of Ebola virus disease laboratory-confirmed cases (EVD+) by chiefdom, Tonkolili district, Sierra Leone, 1 July 2014–30 June 2015

Chiefdom	*N* (%)	Dead *N* (%)	Hospitalised *N* (%)	Incidence rate	Mortality rate	Hospitalisation rate	Inhabitants *N*
Gbonkolenken	76 (16.7)	25 (26.9)	51 (16.8)	127.6	42.0	85.6	59 558
Kafe Simiria	5 (1.1)	2 (2.2)	2 (0.7)	20.1	8.0	8.1	24 901
Kalansogoia	0 (0.0)	0 (0.0)	0 (0.0)	0.0	0.0	0.0	21 873
Kholifa Mabang	3 (0.7)	0 (0.0)	3 (1.0)	17.7	0.0	17.7	16 959
Kholifa Rowalla	126 (27.8%)	29 (31.2)	71 (23.4)	209.0	48.1	117.8	60 289
Kunike	77 (17.0)	5 (5.4)	61 (20.1)	118.3	7.7	116.7	65 105
Kunike Barina	6 (1.3%)	1 (1.1)	2 (0.7)	34.3	5.7	11.4	17 502
Malal Mara	8 (1.8)	3 (3.2)	6 (2.0)	39.8	14.9	29.8	20 117
Sambaia Bendugu	0 (0.0)	0 (0.0)	0 (0.0)	0.0	0.0	0.0	28 503
Tane	51 (11.2%)	8 (8.6)	34 (11.2)	191.3	30.0	127.6	26 654
Yoni	93 (20.5%)	19 (20.4)	66 (21.8)	81.6	16.7	57.9	113 922
other districts (imported)	9 (2.0)	1 (1.1)	7 (2.3)	N.e.	N.e.	N.e.	N.e.
Total	454 (100)	93 (100)	303 (100)	99.7	20.4	66.5	455 383

N.e., not estimable.

In two chiefdoms (Kalansogoia, Sambaia Bendugu), no EVD+ cases were reported. Globally, 9 (2.0%) EVD+ cases were imported from other Sierra-Leonean districts (five Bombali, one Kono, two Western Area Urban and one Western Area Rural). Out of the 454 EVD+ cases, 93 died, determining an EVD+ case fatality ratio (CFR) of 24.5% (range: 0.0% in Kholifa Mabang (*n* = 0/3) to 40% in Kafe Simiria (*n* = 2/5)).

The analysis of key time periods among EVD+ cases showed a median interval from symptom onset to death of 6.0 days (range: 0–20); a median interval form symptom onset to hospitalisation of 3.0 days (range: 0–21) and a median incubation period of 11 days (range: 4–17).

### Risk factors and symptoms associated with EVD+

Table 3 shows the risk factors associated with EVD+.

**Table 3.** Risk factors associated with Ebola virus disease. Univariate (row *χ*^2^) and multivariable analyses (EVD+ *vs*. EVD−). Tonkolili District, Sierra Leone, 1 July 2014–30 June 2015 (*n* = 3798)

Factors		EVD+ (*n* = 454)	EVD− (*n* = 3344)	Total (*n* = 3798)	Univariate analysis	Multivariable analysis^d^
		N (%)	N (%)	N	OR (95% CI)	OR (95% CI)
Gender	Male	197 (10.3)	1719 (89.7)	1916	0.72 (0.59–0.88)^a^	
	Female	257 (13.7)	1625 (86.3)	1882	1.38 (1.13–1.68)^a^	Ref
Age group	<6 years	25 (2.1)	1188 (97.9)	1213	0.11 (0.07–0.16)^a^	Ref
	6–15 years	52 (20.0)	208 (80.0)	260	1.95 (1.41–2.69)^a^	
	16–30 years	160 (19.3)	672 (80.7)	832	2.16 (1.75–2.77)^a^	1.02 (1.00–1.03)^b^
	>30 years	217 (14.5)	1276 (75.5)	1493	1.48 (1.22–1.81)^a^	
Chiefdom of residence	Gbonkolenken	76 (10.8)	625 (89.2)	701	0.87 (0.67–1.13)^c^	
	Kafe Simiria	5 (4.5)	107 (95.5)	112	0.33 (0.13–0.83)^b^	
	Kalansogoia	0 (0.0)	125 (100.0)	125	N.e.	N.e.
	Kholifa Mabang	3 (2.7)	108 (97.3)	111	0.19 (0.06–0.63)^a^	
	Kholifa Rowalla	126 (16.1)	656 (83.9)	782	1.57 (1.26–1.96)^a^	Ref
	Kunike	77 (20.9)	291 (79.1)	368	2.14 (1.63–2.81)^a^	
	Kunike Barina	6 (3.3)	175 (96.7)	181	0.24 (0.10–0.55)^a^	
	Malal Mara	8 (5.9)	127 (94.19)	135	0.45 (0.22–0.93)^b^	
	Sambaya Bendugu	0 (0.0)	30 (100.0)	30	N.e.	N.e.
	Tane	51 (18.6)	223 (81.4)	274	1.77 (1.28–2.44)^a^	
	Yoni	93 (12.2)	668 (87.8)	761	1.03 (0.80–1.31)^c^	
	Outside districts	9 (4.1)	209 (95.9)	218	0.30 (0.15–0.59)^a^	
Occupation	Health care worker	11 (32.49	23 (67.6)	34	3.58 (1.73–7.40)^a^	0.90 (0.86–0.94)^a^
	Other occupation	443 (11.8)	3321 (88.2)	3764	0.28 (0.14–0.58)^a^	Ref
Contact with EVD	Yes	424 (91.2)	41 (8.8)	465	7.39 (3.51–15.05)^a^	2.47 (2.44–2.50)^a^
	No	30 (0.1)	3303 (99.1)	3333	0.61 (0.32–0.77)^a^	Ref
Funeral attendance	Yes	45 (11.2)	358 (88.8)	403	1.09 (0.78–1.51)^c^	1.02 (1.01–1.04)^a^
	No	409 (12.0)	2986 (88.0)	3395	0.91 (0.66–1.27)^c^	Ref

EVD+, Ebola laboratory-confirmed cases; EVD−, Ebola laboratory-excluded cases; OR, odds ratio; N.e., not estimable; Ref, reference value.

a *P* < 0.01.

b *P* < 0.05.

c *P* > 0.05.

d Stepwise backward elimination at 10% level (only significant variables listed).

At the multivariable analysis, no difference was found by gender and chiefdom of residence; whereas, those aged 16–30 years had a higher odd of being EVD+ (OR 1.02; 95% CI 1.00–1.03; *P* < 0.05). In comparison to other occupations, HCWs were 10% less likely to become EVD+ (OR 0.90; 95% CI 0.86–0.94; *P* < 0.01). Having had a contact with an EVD+ or suspected/probable case increased the odds of being EVD+ (OR 2.47; 95% CI 2.44–2.50; *P* < 0.01), as well as attending a funeral and touching/carrying the body (OR 1.02; 95% CI 1.01–1.04; *P* < 0.01).

### Risk factors and symptoms associated with death among EVD+cases

Table 4 shows the risk factors (section A) and symptoms (section B) associated with death among EVD+ cases.

**Table 4.** Risk factors (section A) and symptoms (section B) associated with death among EVD+ cases

Section A: risk factors associated with death among EVD+ cases						
Factors		EVD+ dead (*n* = 93)	EVD+ alive (*n* = 361)	Total (*n* = 454)	Univariate analysis	Multivariable analysis^d^
		N (%)	N (%)	N	OR (95% CI)	OR (95% CI)
Gender	Male	44 (22.3)	153 (77.7)	197	0.81 (0.51–1.29)^c^	
	Female	49 (19.1)	208 (80.9)	257	1.22 (0.77–1.92)^c^	Ref
Age group	<6 years	7 (28.0)	18 (72.0)	25	1.55 (0.63–3.83)^c^	Ref
	6–15 years	12 (23.1)	40 (76.9)	52	0.88 (0.45–1.73)^c^	
	16–30 years	30 (18.8)	130 (81.3)	160	0.85 (0.52–1.37)^c^	
	>30 years	44 (20.3)	173 (79.7)	217	0.98 (0.62–1.54)^c^	
Chiefdom of residence	Gbonkolenken	25 (32.9)	51 (67.1)	76	2.23 (1.29–3.85)^a^	
	Kafe Simiria	2 (40.0)	3 (60.0)	5	2.62 (0.43–15.92)^c^	
	Kalansogoia	0 (0.0)	0 (0.0)	0	N.e.	N.e.
	Kholifa Mabang	0 (0.0)	3 (100.0)	3	N.e.	N.e.
	Kholifa Rowalla	29 (23.0)	97 (77.0)	126	1.23 (0.75–2.02)^c^	Ref
	Kunike	5 (6.5)	72 (93.5)	77	0.22 (0.08–0.58)^a^	0.22 (0.08–0.44)^a^
	Kunike Barina	1 (16.7)	5 (83.3)	6	0.77 (0.08–6.70)^c^	
	Malal Mara	3 (37.5)	5 (62.5)	8	2.37 (0.55–10.11)^c^	
	Sambaya Bendugu	0 (0.0)	0 (0.0)	0	N.e.	N.e.
	Tane	8 (15.7)	43 (84.3)	51	0.69 (0.31–1.53)^c^	
	Yoni	19 (20.4)	74 (79.6)	93	0.99 (0.56–1.75)^c^	
	Outside districts	1 (11.1)	8 (88.9)	9	0.48 (0.05–3.88)^c^	
Occupation	Health care worker	3 (27.3)	8 (72.7)	11	1.47 (0.38–5.66)^c^	
	Other occupation	90 (20.4)	353 (79.6)	443	0.65 (0.18–2.61)^c^	Ref
Hospitalisation	Yes	54 (17.8)	249 (82.2)	303	0.62 (0.39–0.89)^b^	
	No	39 (25.8)	112 (74.2)	151	1.60 (1.06–2.56)^b^	Ref
Symptom onset to hospitalisation	⩾10 days	1 (3.1)	31 (96.9)	32	0.13 (0.01–0.89)^b^	0.08 (0.00–0.41)^b^
	3–9 days	19 (12.8)	130 (87.2)	149	0.49 (0.27–0.81)^b^	0.38 (0.20–0.70)^a^
	⩽2 days	34 (27.9)	88 (72.1)	122	3.11 (1.68–5.72)^a^	Ref

Section B: symptoms associated with death among EVD+ cases						
Factors		EVD+ dead (*n* = 93)	EVD+ alive (*n* = 361)	Total (*n* = 454)	Univariate analysis	Multivariable analysis^d^
		N (%)	N (%)	No.	OR (95% CI)	OR (95% CI)
Gender	Male	44 (22.3)	153 (77.7)	197	0.81 (0.51–1.29)^c^	
	Female	49 (19.1)	208 (80.9)	257	1.22 (0.77–1.92)^c^	Ref
Age group	<6 years	7 (28.0)	18 (72.0)	25	1.55 (0.63–3.83)^c^	Ref
	6–15 years	12 (23.1)	40 (76.9)	52	0.88 (0.45–1.73)^c^	
	16–30 years	30 (18.7)	130 (81.3)	160	0.85 (0.52–1.37)^c^	
	>30 years	44 (20.3)	173 (79.7)	217	0.98 (0.62–1.54)^c^	
Symptoms	Anorexia	55 (22.4)	191 (77.6)	246	1.28 (0.81–2.04)^c^	
	Headache	55 (16.3)	283 (83.7)	338	0.39 (0.24–0.64)^a^	0.40 (0.25–0.66)^a^
	Joint/muscle pain	45 (17.0)	219 (83.0)	264	0.60 (0.38–0.96)^b^	
	Chest pain	32 (20.9)	121 (79.1)	153	1.04 (0.64–1.68)^c^	
	Hiccups	5 (26.3)	14 (73.7)	19	1.40 (0.49–4.01)^c^	
	Abdominal and stomach pain	53 (17.3)	254 (82.7)	307	0.55 (0.34–0.89)^b^	0.56 (0.34–0.91)^b^
	Fatigue and weakness	75 (21.5)	274 (78.5)	349	1.32 (0.75–2.33)^c^	
	Diarrhoea	37 (18.8)	160 (81.2)	197	0.83 (0.52–1.32)^c^	
	Vomit	39 (18.3)	174 (81.7)	213	0.77 (0.49–1.23)^c^	
	Fever	93 (20.59	361 (79.5)	454	N.e.	N.e.
	Difficult breathing	20 (19.4)	83 (80.6)	103	0.91 (0.52–1.59)^c^	
	Difficult swallowing	8 (20.5)	31 (79.5)	39	1.00 (0.44–2.25)^c^	
	Sore throat	5 (26.3)	14 (73.7)	19	1.40 (0.49–4.01)^c^	
	Jaundice	1 (7.1)	13 (92.9)	14	0.29 (0.03–2.25)^c^	
	Conjunctivitis	13 (20.0)	52 (80.0)	65	0.96 (0.50–1.86)^c^	
	Rash	4 (40.0)	6 (60.0)	10	2.65 (0.73–9.62)^c^	
	Cough	3 (16.7)	15 (83.3)	18	0.76 (0.21–2.71)^c^	
	Unexplained bleeding	77 (21.7)	278 (78.3)	355	1.43 (0.79–2.59)^c^	

Univariate (row *χ*^2^) and multivariable analyses (EVD+ dead *vs*. EVD+ alive cases). Tonkolili district, Sierra Leone, 1 July 2014–30 June 2015 (*n* = 454).

EVD+, Ebola laboratory-confirmed cases; OR, odds ratio; N.e., not estimable; Ref, reference value.

a *P* < 0.01.

b *P* < 0.05.

c *P* > 0.05.

d Stepwise backward elimination at 10% level (only significant variables listed).

At the multivariable analysis, only two risk factors remained statistically significant: EVD+ cases from Kunike chiefdom had a lower odds of death (OR 0.22; 95% CI 0.08–0.44; *P* < 0.01); whilst the odd of death was inversely proportional to the duration of the time interval from symptom onset to hospitalisation: the longer was this interval, the lower was the risk of death (⩾10 days; OR 0.08; 95% CI 0.00–0.41; *P* < 0.05). Among symptoms, only headache (OR 0.40; 95% CI 0.25–0.66; *P* < 0.01) and abdominal/stomach pain (OR 0.56; 95% CI 0.34–0.91; *P* < 0.05) were statistically significantly correlated, with a protective effect *vs.* the odds of death.

### Factors and symptoms associated with hospitalisation among EVD+ cases

Table 5 shows the factors (section A) and symptoms (section B) associated with hospitalisation among EVD+ cases.

**Table 5.** Factors (section A) and symptoms (section B), associated with hospitalisation among EVD+ cases

Section A: factors associated with hospitalisation among EVD+ cases						
Factors		EVD+ hospitalised (*n* = 303)	EVD+ non-hospitalised (*n* = 151)	Total (*n* = 454)	Univariate analysis	Multivariable analysis^d^
		*N* (%)	*N* (%)	*N*	OR (95% CI)	OR (95% CI)
Gender	Male	141 (71.6)	56 (28.4)	197	1.47 (0.99–2.20)^c^	1.60 (1.10–2.39)^a^
	Female	162 (63.0)	95 (37.0)	257	0.67 (0.45–1.01)^c^	Ref
Age group	<6 year	11 (44.0)	14 (56.0)	25	0.37 (0.16–0.82)^a^	Ref
	6–15 years	36 (69.2)	16 (30.8)	52	1.14 (0.61–2.12)^c^	2.91 (1.06–8.29)^b^
	16–30 years	98 (61.3)	62 (38.8)	160	0.69 (0.46–1.03)^c^	
	>30 years	158 (72.8)	59 (27.2)	217	1.70 (1.14–2.53)^a^	3.55 (1.48–8.72)^a^
Chiefdom of residence	Gbonkolenken	50 (65.8)	26 (34.2)	76	0.95 (0.56–1.59)^c^	
	Kafe Simiria	2 (40.0)	3 (60.0)	5	0.32 (0.05–1.98)^c^	
	Kalansogoia	0 (0.0)	0 (0.0)	0	N.e.	N.e
	Kholifa Mabang	3 (100.0)	0 (0.0)	3	N.e.	N.e.
	Kholifa Rowalla	73 (57.9)	53 (42.1)	126	0.58 (0.38–0.89)^b^	Ref
	Kunike	59 (76.6)	18 (23.49)	77	1.78 (1.04–3.15)^b^	2.34 (1.23–4.57)^b^
	Kunike Barina	2 (33.3)	4 (66.7)	6	0.24 (0.04–1.34)^c^	
	Malal Mara	7 (87.5)	1 (12.5)	8	3.54 (0.43–29.09)^c^	
	Sambaya Bendugu	0 (0.0)	0 (0.0)	0	N.e.	N.e.
	Tane	33 (64.7)	18 (35.3)	51	0.90 (0.49–1.66)^c^	
	Yoni	67 (72.0)	26 (28.0)	93	1.36 (0.82–2.25)^c^	2.06 (1.14–3.79)^b^
	Outside districts	7 (77.8)	2 (22.2)	9	1.76 (0.36–8.58)^c^	
Occupation	Health care worker	9 (81.8)	2 (18.2)	11	2.28 (0.48–10.68)^c^	
	Other occupation	294 (66.4)	149 (33.6)	443	0.43 (0.09–2.06)^c^	Ref
Contact with EVD	Yes	290 (68.4)	134 (31.6)	424	2.83 (1.33–5.99)^a^	2.45 (1.12–5.48)^b^
	No	13 (43.3)	17 (56.7)	30	0.35 (0.16–0.748)^a^	Ref
Funeral attendance	Yes	29 (64.4)	16 (35.6)	45	0.89 (0.46–1.70)^c^	
	No	274 (67.0)	135 (33.0)	409	1.12 (0.58–2.13)^c^	Ref

Section B: symptoms associated with hospitalisation among EVD+ cases						
Factors		EVD+ hospitalised (*n* = 303)	EVD+ non-hospitalised (*n* = 151)	Total (*n* = 454)	Univariate analysis	Multivariable analysis^d^
		N (%)	N (%)	N	OR (95% CI)	OR (95% CI)
Gender	Male	141 (71.6)	56 (28.4)	197	1.47 (0.99–2.20)^c^	
	Female	162 (63.0)	95 (37.0)	257	0.67 (0.45–1.01)^c^	Ref
Age group	<6 year	11 (44.0)	14 (56.0)	25	0.37 (0.16–0.82)^a^	Ref
	6–15 years	36 (69.2)	16 (30.8)	52	1.14 (0.61–2.12)^c^	
	16–30 years	98 (61.3)	62 (38.7)	160	0.69 (0.46–1.03)^c^	2.34 (1.56–5.35)^a^
	>30 year	158 (72.8)	59 (27.3)	217	1.70 (1.14–2.53)^a^	3.41 (1.43–8.39)^b^
Symptoms	Anorexia	166 (67.5)	80 (32.5)	246	1.07 (0.72–1.59)^c^	
	Headache	222 (65.7)	116 (34.3)	338	0.82 (0.52–1.30)^c^	
	Joint/muscle pain	176 (66.7)	88 (33.3)	264	0.99 (0.66–1.47)^c^	
	Chest pain	14 (73.7)	5 (26.3)	19	1.30(0.85–1.98)^c^	
	Hiccups	108 (70.6)	45 (29.4)	153	1.41 (0.25–4.00)^c^	
	Abdominal and stomach pain	209 (68.1)	98 (31.9)	307	1.20 (0.79–1.81)^c^	
	Fatigue and weakness	244 (69.9)	105 (30.1)	349	1.81 (1.15–2.87)^b^	
	Diarrhoea	135 (68.5)	62 (31.5)	197	1.15 (0.77–1.71)^c^	
	Vomit	152 (71.4)	61 (28.69)	213	1.48 (1.09–2.20)^b^	
	Fever	303 (66.7)	151 (33.3)	454	N.e.	N.e.
	Difficult breathing	71 (68.9)	32 (31.1)	103	1.13 (0.71–1.82)^c^	5.43 (1.89–23.0)^b^
	Difficult swallowing	36 (92.3)	3 (7.7)	39	6.65 (2.01–11.97)^a^	
	Sore throat	17 (89.5)	2 (10.5)	19	4.42 (2.01–9.42)^b^	
	Jaundice	14 (100.0)	0 (0.0)	14	N.e.	N.e.
	Conjunctivitis	54 (83.1)	11 (16.9)	65	2.76 (1.39–5.45)^a^	
	Rash	8 (80.0)	2 (20.0)	10	2.02 (0.42–9.63)^c^	
	Cough	12 (66.7)	6 (33.3)	18	0.99 (0.36–2.70)^c^	
	Unexplained bleeding	248 (69.9)	107 (30.1)	355	1.85 (1.17–2.92)^b^	1.64 (1.10–2.65)^b^

Univariate (row *χ*^2^) and multivariable analyses (EVD+ hospitalised *vs*. EVD+ non-hospitalised). Tonkolili district, Sierra Leone, 1 July 2014–30 June 2015 (*n* = 454).

EVD+, Ebola laboratory-confirmed cases; OR, odds ratio; N.e., not estimable; Ref, reference value.

a *P* < 0.01.

b *P* < 0.05.

c *P* > 0.05.

d Stepwise backward elimination at 10% level (only significant variables listed).

Among factors, at the multivariable analysis, EVD+ male patients (OR 1.60; 95% CI 1.10–2.39; *P* < 0.01), aged 6–15 (OR 2.91; 95% CI 1.06–8.29; *P* < 0.05) and >30 years (OR 3.55; 95% CI 1.48–8.72; *P* < 0.01), were more likely to be hospitalised, as well as EVD+ cases from Kunike (OR 2.34; 95% CI 1.23–4.57; *P* < 0.05) and Yoni chiefdoms (OR 2.06; 95% CI 1.14–3.79; *P* < 0.05) and EVD+ cases reporting a contact with an EVD+ or suspected/probable case (OR 2.45; 95% CI 1.12–5.48; *P* < 0.05).

Among symptoms, EVD+ cases that presented at the HCF/ETC/ETU admission with difficult breathing (OR 5.43; 95% CI 1.89–23.0; *P* < 0.05) and unexplained bleeding (OR 1.64; 95% CI 1.10–2.65; *P* < 0.05) were more likely to be hospitalised.

### Assessment of key-performance indicators for EVD response

Table 6 shows the values of key-performance indicators for EVD response attained in Tonkolili district compared with the WHO/MoH targets.

**Table 6.** Assessment of Word Health Organization and Sierra Leone Ministry of Health indicators for Ebola response in Tonkolili district, Sierra Leone, 1 July 2014–30 June 2015

Indicator	Tonkolili district N (%)	WHO/MoH target
Percentage of alert generated within 1 day from symptoms onset	1141 (25.1)	100%
Number and percentage of health care workers infected	11 (20.7)	0%
Percentage of samples tested within 1 day of collection	2857 (75.2)	100%
Percentage of deaths buried within 1 day ^a^	2841 (97.9)	100%
Percentage of lives alert tested for Ebola virus	1350 (81.8)	100%
Percentage of deaths alert tested for Ebola virus	2448 (84.4)	100%
Percentage of reported community deaths that were tested for Ebola virus	1638 (82.2)	100%
Percentage of new confirmed cases from registered contacts	424 (93.4)	100%
Number of hospitalised within 3 days from symptom onset	1228 (62.8)	100%
Case fatality rate among hospitalised EVD+ cases	54 (17.8)	<60%
Number of Ebola survivors by gender, age group, chiefdom	Reported	Reported
Number of contacts traced per EVD+ case	Reported	Reported

a All burials (safe and dignified burials and not).

WHO, World Health Organization; MoH, Ministry of Health; EVD+, Ebola laboratory-confirmed cases.

The median interval from symptom onset to alert generation was 4 days (range: 0–28), with 25.1% of alerts generated within 1 day from symptom onset. Among the 53 alerts related to HCWs, 11 (20.7%) resulted EVD+. Out of 4550 alerts, 3798 (83.5%) were laboratory investigated (81.8% from alive and 84.4% from dead patients). The median interval from alert generation to sample collection was 0 days (range: 0–20), with 79.4% of samples collected within 1 day from the alert; while the median interval from sample collection to sample testing was 1 day (range 0–20), with 75.2% of samples tested within 1 day from collection. The median interval from death-to-burial was 0 days (range: 0–9), with 97.9% buried within 1 day from death. Furthermore, 62.8% of hospitalisations (*n* = 1228/1954) occurred within 3 days from symptom onset, and the CFR among EVD+ hospitalised cases was 17.8%. Among the 454 EVD+ cases, 424 (93.4%) were already recorded in a contacts-tracing list.

Finally, according to WHO/MoH targets, the information on the number of EVD+ survivors (by gender, age group, chiefdom) was reported in the Tonkolili VHF surveillance system (Table 4, section A), as well as the number of contacts traced per EVD+ case, with an average of 21 individuals (range: 11–79).

Logistic regression model assessing the achievement of indicators over the time and by chiefdom (Table 7) showed gaps in the last trimester of the study (April–June 2015), when the odds that an alert was generated within 1 day from symptoms onset was 40% lower (OR 0.61; 95% CI 0.43–0.87; *P* < 0.05); whilst no differences were observed over the time for the other indicators, which were all statistically significant above the OR of 1.00.

**Table 7.** Logistic regression model^a^ assessing the correlation between time (trimesters), place (chiefdom) and the achievement of the indicator, Tonkolili district, Sierra Leone, 1 July 2014–30 June 2015

Factors		Indicators					
		Alert generated within 1 day from symptoms onset	Sample tested within 1 day of collection	Lives alert tested for Ebola virus	Deaths alert tested for Ebola virus	Community deaths tested for Ebola virus	Hospitalisation within 3 days from symptom onset
Chiefdom	Gbonkolenken	Ref	Ref	Ref	Ref	Ref	Ref
	Kafe Simiria	1.72 (1.14–2.56)^b^	0.58 (0.37–0.92)^b^	0.91 (0.39–2.35)	0.30 (0.17–0.52)^b^	0.24 (0.12–0.45)^b^	1.99 (1.07–3.92)^b^
	Kalansogoia	1.24 (0.79–1.91)	0.51 (0.33–0.79)^b^	0.35 (0.10–1.40)	0.79 (0.43–1.53)	0.63 (0.31–1.35)	0.74 (0.40–1.40)
	Kholifa Mabang	0.68 (0.40–1.11)	0.93 (0.57–1.59)	0.70 (0.23–2.65)	0.42 (0.24–0.75)^b^	0.41 (0.21–0.81)^b^	1.06 (0.53–2.21)
	Kholifa Rowalla	1.22 (0.97–1.54)	1.13 (0.87–1.48)	0.86 (0.57–1.31)	0.90 (0.59–1.36)	0.98 (0.59–1.62)	1.15 (0.85–1.55)
	Kunike	0.95 (0.70–1.27)	0.4 (0.30–0.53)^b^	1.04 (0.63–1.74)	0.30 (0.20–0.47)^b^	0.24 (0.14–0.38)^b^	0.73 (0.51–1.04)
	Kunike Barina	1.53 (1.07–2.19)^b^	0.78 (0.53–1.18)	0.60 (0.28–1.43)	0.66 (0.39–1.17)	0.39 (0.20–0.76)^b^	1.04 (0.65–1.67)
	Malal Mara	1.47 (0.99–2.16)	0.46 (0.31–0.70)^b^	0.25 (0.11–0.53)^b^	0.26 (0.16–0.42)^b^	0.23 (0.13–0.40)^b^	1.00 (0.54–1.89)
	Other district	1.48 (1.05–2.09)^b^	1.15 (0.77–1.74)	1.01 (0.59–1.76)	1.04 (0.47–2.64)	1.13 (0.25–9.72)	0.91 (0.61–1.36)
	Sambaya Bendugu	1.24 (0.47–2.86)	0.30 (0.14–0.67)^b^	No observations	0.52 (0.20–1.60)	0.27 (0.10–0.91)^b^	0.80 (0.28–2.48)
	Tane	0.71 (0.50–1.00)	0.99 (0.70–1.42)	1.27 (0.71–2.33)	0.66 (0.39–1.11)	0.72 (0.39–1.36)	0.74 (0.49–1.11)
	Yoni	0.99 (0.78–1.26)	0.45 (0.35–0.57)^b^	0.94 (0.57–1.55)	0.56 (0.38–0.81)^b^	0.51 (0.33–0.79)^b^	1.09 (0.78–1.51)
Trimester	July–September 2014	Ref	Ref	Ref	Ref	Ref	Ref
	October–December 2014	4.36 (3.16–6.12)^b^	1.95 (1.36–2.78)^b^	6.47 (4.62–9.10)^b^	1.44 (0.61–3.31)	5.35 (0.73–113.11)	1.87 (1.37–2.55)^b^
	January–March 2015	2.14 (1.54–3.01)^b^	2.93 (2.04–4.2)^b^	4.05 (2.77–5.98)^b^	6.75 (2.86–15.51)^b^	31.79 (4.40–670.49)^b^	3.78 (2.7–5.31)^b^
	April–June 2015	0.61 (0.43–0.87)^b^	2.35 (1.64–3.36)^b^	5.71 (3.53–9.49)^b^	4.55 (1.94–10.39)^b^	25.95 (3.61–546.2)^b^	1.63 (1.15–2.32)^b^

Ref, reference value.

a Adjusted by age group and gender.

b Statistically significant (*P* < 0.05).

Concerning the number of sample tested within 1 day of collection, several chiefdoms had a statistically significant lower odd of reaching the target. The same was observed with the number of deaths occurred at the HCF/ETC/ETU and in the community tested for EBOV, with several chiefdoms showing a statistically significant lower odd of reaching the indicator. Finally, only the chiefdom of Kafe Simiria had a statically significant odd that the hospitalisation occurred within 3 days from symptom onset (OR 1.99; 95% CI 1.07–3.92; *P* < 0.05).

## Discussion

This study presents first whole epi-data on the EVD outbreak in Tonkolili district, showing how the VHF database can be used to identify risk factors and to measure MoH/WHO key-performance indicators for EVD response.

EVD transmission in Tonkolili district began in August 2014 in the chiefdom of Kholifa Rowalla that showed the highest EVD+ IR at the end of the outbreak; but before the implementation of the EVD CEBS/VHF surveillance system in Tonkolili (July 2014), many suspected/probable cases may have never been reported, investigated and tested for EVD, as suggested in the literature [25] and by the fact that the number of alerts increased exponentially with time.

Overall, during the 1-year study period, 454 EVD+ cases were reported by the VHF surveillance system of Tonkolili district, with the last on 15 March 2015. After the study period, other three EVD+ cases occurred in Tonkolili district: on the 24 July 2015 [14], 13 September 2015 and 14 January 2016 [16] leading to the final total of 457 EVD+ cases declared by WHO and Sierra Leone MoH [14, 15].

The monthly EVD+ IR in Tonkolili district and Sierra Leone showed a similar temporal pattern, reaching the peak in October 2014, when the Tonkolili IR was higher than the national IR (400.5 *vs.* 383.1/100 000) [15]. Overall, the Tonkolili district EVD+ IR was lower than the national IR (99.7 *vs.* 122.2/100 000 inhabitants) [10], but differences were observed at chiefdom level with someone not affected (e.g. Kalansogoia and Sambaia Bendugu) and others (e.g. Kholifa Rowalla) showing a higher IR compared with the district and national one. Although the central geographical location of Tonkolili district, the proportion of EVD+ imported cases was low (2%) compared with that reported in other districts (Pujehun 18%; Kono 13%) [26, 27].

Concerning risk factors for EVD+, similarly to other studies [28, 29], no gender-related difference was found. Although contrasting, data from the literature [29, 30] seems to consider at higher risk for EVD+ patients aged 35–44 years, with slight increase or reduction thereafter. In this study, patients aged 16–30 years showed an increased odd (at the limit of statistical significance) of being EVD+; and this is in line with the age-specific incidence of EVD reported during the West Africa outbreak, and probably due to the fact that this age group could be more exposed to care sick people and in funeral preparations [30].

Differently from other studies conducted during the West Africa EVD outbreak [30, 31], HCWs of Tonkolili district had a lower odd of being EVD+ compared with other professional categories, as confirmed by their small proportion among EVD+ cases (*n* = 11/454; 2.4%), one of the lowest recorded in Sierra Leone [32].This finding may have been driven by the impossibility of further disaggregate work categories in the statistical models, and this, together with the unavailability of the information on the type of HCWs, is a limit of this analysis. On the other hand, a study conducted in Sierra Leone [33] showed the absence of EBOV glycoprotein IgG among HCWs compared with other professional categories, suggesting a lower exposition or a greater protection to exposition, as also reported in a study conducted in Guinea [34].

History of contact with confirmed or suspected/probable EVD cases increased the odd of being EVD+ in this study, being reported by 93.4% (*n* = 424/454) of EVD+ cases, similarly to what observed in smaller studies from other districts of Sierra Leone [28, 34]. At the multivariable analysis, attending a funeral and touching/carrying the body was another factor (at the limit of statistical significance) increasing the odd of EVD+, as reported in a meta-analysis on Ebola-transmission risk factors [28] and in a study from Moyamba district in Sierra Leone [35].

The EVD+ mortality rate in Tonkolili district was lower than that of Sierra Leone (20.4 *vs.* 50.4/100 000 inhabitants), with all the chiefdoms reporting values below the national morality rate [3]. Also the EVD+ CFR was lower in Tonkolili district compared with the one at national level (24.5% *vs.* 41.2%), and with the CFR of other Sierra Leonean districts (Pujehun 85.7%, Kono 64%, Moyamba 58%) [26, 27, 36].

Concerning risk factors for death among EVD+ cases, no difference was found by gender and age group. In a nation-wide retrospective study conducted in Guinea, the age was the only factor independently associated with EVD-related mortality [37], as in another smaller study from Sierra Leone [38]; no gender-related differences were instead found in both studies, differently from a study performed at West Africa level, where male sex was associated with death [39]. The observation that the longer was the interval from symptom onset to hospitalisation the lower was the odd of death may be related to symptoms’ severity, possibly because more severe cases may seek medical care earlier [40]. Abdominal pain and headache were symptoms associated with a protective effect *vs.* the odd of death; this could be explained by the fact that patients reporting these two symptoms had not more severe clinical presentation, and on the other hand, patients with severe symptoms might have not been able to report these two mild symptoms.

Although in the present study, the hospitalisation was not associated with death, as instead reported in the literature [41], EVD+ cases from Kunike-chiefdom showed a protection *vs.* the odd of death and also had more probability to be hospitalised, suggesting a protective effect of the hospitalisation. On this regard, in Tonkolili district, there were only two ETCs: one was at the Magburaka hospital (Bombalì District), and one was an Médecins Sans Frontières’ (MSF) ETC opened in December 2014 in Kholifa Rowalla chiefdom [42]. Kunike was not the closer chiefdom to these ETCs, and Kholifa Rowalla had not the highest hospitalisation rate, suggesting no effect of the ETC's proximity on the chance to be hospitalised and on the risk of death.

Also EVD+ cases with history of contact with EVD+ or suspected/probable cases and with unexplained bleeding were more likely to be hospitalised, suggesting an effective implementation of the contact-tracing process. Findings related to EVD+ hospitalisation cannot be compared with others, due to the lack of these data in the literature.

Observed key-time periods were similar with those reported in the literature. The median EVD incubation period was 11 days, in line with what reported from Pujehun district (10 days) [25] and from a meta-analysis (10.3 days) [27]. The observed median intervals from symptom onset to death and to hospitalisation were 6 and 3 days, respectively, similarly to what reported from Pujehun (5.7 and 4.5 days, respectively) [25].

Globally, the assessment of WHO/MoH key-performance indicators for EVD response found gaps mainly related to the interval from symptom onset to alert generation in particular in the last trimester of the study, and this is probably due to the exponential increase in the number of alerts over the time. Acceptable performances were observed once the alert is generated, even if 25% of samples were not collected <1 day from the alert.

A comparison of these indicators with those attended in other districts is not possible due to the lack of data in the literature.

Albeit beyond the objectives of the study, it is noteworthy to be underlined that the CFR among EVD or suspected/probable cases was extremely high (68.5%, *n* = 2806/4096), but data on cause of death were not available. This high mortality is strictly related to the chronic weakness of health systems of resource-limited countries in Africa that can easily collapse when epidemics, as the EVD outbreak, strikes [43].

A limitation of this study is related to the broad age groups used in order to improve the stability of the multivariable model, though this has not prevented to identify specific age group at increased risk for EVD+ in the district. As reported in the literature [44], another limitation is due to the fact that the number of true cases of EVD may be ⩾2.5 times that of reported cases and consequently risk factors may be different or have a different weight in the overall picture of the outbreak into the district.

Despite these limitations and differently from the literature [9], the data quality and data completeness of the Tonkolili district VHF database, as indicated, for example, by the fact that all the 454 EVD+ cases had a known risk factor, allowed designing a clear picture of the EVD outbreak, identifying gaps in the response and using for the first time EVD– alerts as controls to identify risk factors; EVD– alerts work as controls of high-quality because they are generated from the same source of EVD+ alerts.

In conclusion, this study also outlined lessons learned: future EVD preparedness and response plans for Tonkolili district should include a chiefdom-vision approach and community-based strategy of social-mobilisation activities targeting Ebola knowledge-attitudes-practice (a) during funeral attendance (b) to avoid contact with EVD+ or suspected/probable cases and (c) to increase awareness on EVD symptoms, in order to reduce the delays between symptom onset and treatment seeking and so the delays between symptom onset and alert generation that consequently affect the promptness of the outbreak response.

## Supplementary material

For supplementary material accompanying this paper visit https://doi.org/10.1017/S0950268819000177. click here to view supplementary material
