# Supplementary figures and images for: The Ebola virus disease outbreak in Tonkolili district, Sierra Leone: a retrospective analysis of the Viral Haemorrhagic Fever surveillance system, July 2014–June 2015
Source: Epidemiol Infect. 2019 Feb 26;147:e103. doi: 10.1017/S0950268819000177 (PMC6518516; doi:10.1017/S0950268819000177)

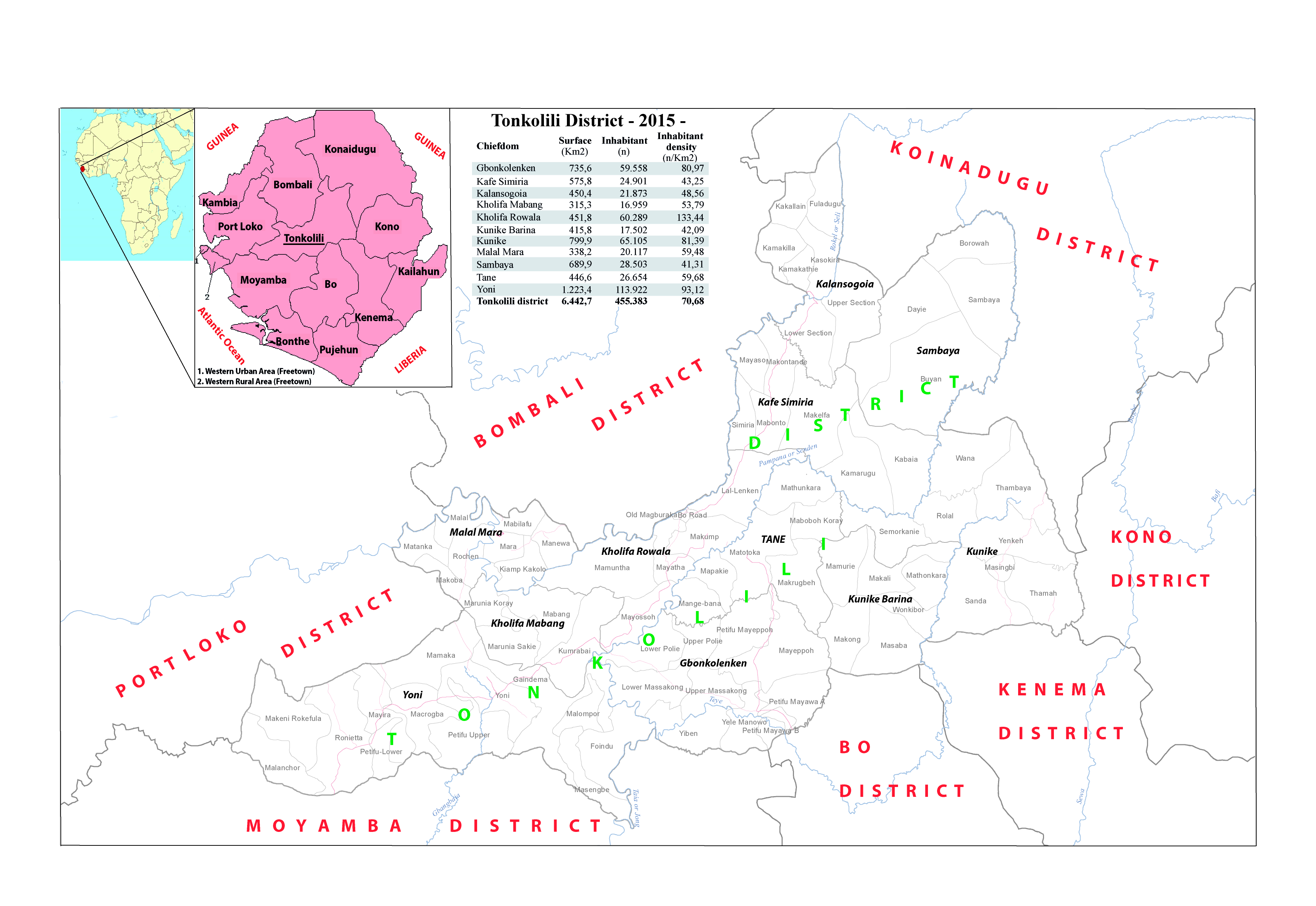

Supplement: Supplementary file 1 [file S0950268819000177sup001.zip › FigS1.tif]
